# Categorization of adverse drug reactions in electronic health records

**DOI:** 10.1002/prp2.550

**Published:** 2020-04-17

**Authors:** Caroline Foreman, William B. Smith, Gillian E. Caughey, Sepehr Shakib

**Affiliations:** ^1^ Immunology Department Royal Adelaide Hospital Adelaide Australia; ^2^ Discipline of Pharmacology Adelaide Medical School University of Adelaide Adelaide Australia; ^3^ Department of Clinical Pharmacology Royal Adelaide Hospital Adelaide Australia; ^4^ School of Pharmacy and Medical Sciences Division of Health Sciences University of South Australia Adelaide Australia

**Keywords:** adverse drug reactions, drug allergy, electronic health record

## Abstract

The purpose of this study was to evaluate the quality of adverse drug reaction (ADR) documentation in a state‐wide electronic health record (EHR), and to assess the impact of the interface design on documentation accuracy and ability to provide decision support. Data were extracted from 43 011 unique records in a state‐wide electronic health record in South Australia, Australia. Information obtained included ADR coding as allergy or intolerance, allergen name, reaction, and occupation of those entering data. Categorization into drug allergy or intolerance was assessed for accuracy. Reactions were entered predominantly by nurses (60.1%), also by doctors (31.0%) and pharmacists (6.1%). Of 27 314 reactions, 86.5% were coded as allergy and 13.5% as intolerance. The majority (78.2%) described reactions to drugs (as opposed to food, environmental or contact allergens), predominantly chosen from the drug database (96.4%). Many entries used free text for the reaction description (27.4%). Terms found in the predefined list under the allergy heading were more likely to be categorized as allergy, even when the mechanism was pharmacological intolerance. Only 45.1% (n = 1671/3705) of reactions consistent with intolerance (eg, “nausea,” “diarrhea”) were correctly categorized as such, although categorization by pharmacists was more accurate (*P* < .0001). These data suggest that ADR categorization as allergy or intolerance is influenced by the EHR design. The obligatory classification of ADRs into allergy or intolerance was not well understood and does not appear to have practical benefit.

**What is already known about this subject**

- Computerized drug alert checking has been shown to reduce medication‐related harm.
- Type A adverse reactions occur because of susceptibility to the adverse pharmacological actions of a drug (intolerance), have diverse manifestations, and are the most common type of ADR.
- Type B adverse reactions are due to immunological mechanisms, tend to have characteristic manifestations, and are unpredictable unless they have occurred previously.

**What this study adds**

- Categorization as allergy or intolerance appears to be influenced by the design of the EHR so that the majority of ADRs were categorized as allergy, as this was the default value, even for obvious intolerances.
- Despite the availability of a comprehensive set of reaction descriptors in a checklist, free text fields were used to describe > 25% of reactions and the majority of opioid ADRs.
- The obligatory classification of ADRs into allergy or intolerance provides no practical benefit and may be counterproductive.

## 1 INTRODUCTION

A patient history of an adverse drug reaction (ADR) predicts an increased risk of another adverse reaction on subsequent exposure to the same and related drugs and is a prescribing hazard. Computerized Physician Order Entry with decision support has been shown to reduce medication‐related harm,^1^, ^2^, ^3^ and drug alert checking is an important part of this benefit.^1^, ^4^ Unlike other decision support tools, ADR alerting is highly reliant on the information documented at the time of data input. Cross‐sensitivity checking is only possible if the drug causing the adverse reaction is coded correctly, and tiering of alerts is only possible if reaction types are coded accurately.

An electronic health record (EHR) system has recently been rolled out across South Australian (SA) public hospitals. ADRs are entered via an “allergies” module that includes; recording of adverse reactions to drugs, foods, contact factors, environment, or blood products. All EHR users, including allied health practitioners, students and clerical staff, may enter ADR information. Allergy recording or checking is prompted every time that any user submits an order (prescription, dietary, pathology, radiology) through the system. The type of causative substance (drug, food, contact, or other) is first chosen, which then determines the subsequent list (list of drug names, or list of foods, etc.) from which the actual substance is chosen. It is then mandatory to choose a reaction category of allergy or intolerance before entry of further details, with allergy as the default option. Reaction types are selected via check box lists, with different predetermined reaction lists for allergy and intolerance. Substance lists and reaction type lists allow the selection of “other” and the entry of free text, but this results in an inability to carry out system‐generated alert checking.

When classifying an ADR according to mechanism, the term “allergy” refers to an immunologically mediated reaction (classically type B ADR), and “intolerance” to type A reactions or side effects.^5^ We recently performed a scenario‐based study to evaluate the understanding of SA health care workers (HCW) in the categorization of ADRs as allergy or intolerance^6^ We determined that the accuracy of this choice was poor and is not influenced by the professional background. Although this study assessed the understanding of clinicians regarding ADR documentation, the accuracy of actual ADR documentation in the EHR and the influence of the interface design on this is not known. The aim of this study was to evaluate the quality of ADR documentation in a state‐wide EHR, and assess the potential impact of the interface design on documentation accuracy and ability to provide decision support to improve patient care.

## 2 MATERIALS AND METHODS

Ethics approval for this study was obtained from the South Australian Health Ethics Committee.

We conducted a cross‐sectional retrospective review of ADR documentation in the newly implemented EHR (Enterprise Patient Administration System (EPAS); a modification of the Allscripts Sunrise System (Allscripts)) at three public hospitals in South Australia between August 2013 and January 2015. These included two metropolitan hospitals (Noarlunga Public Hospital and Repatriation General Hospital) and one regional hospital (Port Augusta Hospital and Regional Health Services). The services provided include acute inpatient care for adults, maternity and pediatrics, as well community and outpatient services. The entire patient dataset was examined, all allergy records were extracted, including explicit reports of “no known allergies,” “unknown allergy status,” or a specific allergy or intolerance to a drug or other substance; the reaction category, allergy or intolerance; the coded causative drug; the nature of the reaction, and severity and descriptive information entered as free text; and the occupation of the health care worker (HCW) entering the information. No patient identification or demographic information was extracted.

We assessed how ADRs were categorized, that is, as “allergy” or “intolerance”; whether the reactions were entered using the drop down lists (which could be coded) or free text; and whether this was influenced by the profession of the clinician entering these reports. Common examples of reaction types consistent with allergy were identified using the search terms: “rash,” “anaphylaxis,” “angioedema,” and “urticaria.” We defined the following common reactions as being unlikely to be mediated by immunological mechanisms and therefore intolerance: “nausea,” “gastrointestinal upset,” “diarrhea,” “cramps,” “constipation,” and “headache.”

Data on some specific drug or reaction scenarios, including angiotensin converting enzyme (ACE)‐inhibitor‐induced angioedema, anaphylaxis, Stevens Johnson Syndrome (SJS), statin‐associated myalgia, NSAID‐induced urticaria or rash, opiate reactions which did not have characteristics of allergy, and headache were extracted. These were compared with scenarios in our previous ADR understanding study,^6^ which allowed us to determine the impact of the user interface in the documentation of reactions.

The data were analyzed using Excel (Microsoft). Pearson's Chi Square test was used to compare proportions with a *P*‐value of < .05 as significant. Fisher's exact test was used to compare categorization into allergy/intolerance dependent on survey or EHR.

## 3 RESULTS

**Figure 1:**
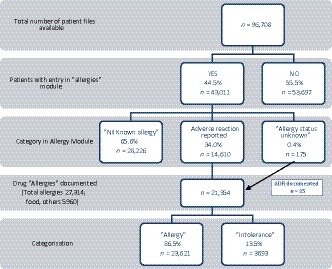
Study population and stratification depending on categorization of allergy alert

**Figure 2:**
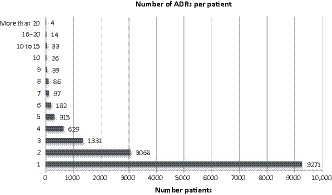
Frequency of number of reactions documented for each patient

Of the 96 708 patients entered into EPAS, 44.5% had an entry in the “allergies” module, with the majority of these reporting nil known allergies (65.6%) and 34% of patients having one or more documented adverse reactions. 175 patients had “allergy status unknown” including 15 instances where an actual adverse drug reaction was documented in this field. 15.6% of all patients had a total of 27 314 reactions documented, categorized as allergy (86.5%) or intolerance (13.5%) (Figure 1). The number of documented reactions per patient ranged from one to 33. The frequency of the number of reactions documented per patient is displayed in Figure 2, with the vast majority (12 339/15 095, 81.7%) having only one or two documented reactions.

Of the 27 314 reactions documented, 21 354 (78.2%) were for drugs and 5960 (21.8%) were for others, predominantly foods. The commonest class of medications documented were penicillins (23.5%), followed by opioids (22.5%) and other nonpenicillin antibiotics (17.4%). A total of 473 reactions (2.2% of total) were entered as “sulfur” drugs. 5.2% of the total patient cohort were labelled penicillin‐allergic.

In the majority of entries, the causative agent was chosen from the drop down list of drug names; but for 974 (3.6%) of the reactions, including 87 drugs, the allergen chosen was “other”; and the drug name was entered as free text; therefore, not permitting drug allergy crosschecks. In the majority of these cases the drug names were actually available in the drop down list of allergens. In these cases, the clinician performing the data entry had chosen the “other” category and free text option instead. This was often due to misspelling of drug name or use of a colloquial term such as “local anesthetic,” “muscle relaxant for anesthetic,” and “X‐ray dye.”

In the majority of entries, the reaction type was chosen from the check box list that was provided after the choice of allergy or intolerance had been made, but a total of 7,481 reactions (27.4% of total) had only a free text description of the adverse reaction that was entered after selecting the reaction descriptor “other.” A greater proportion of records categorized as “allergy” had free text reaction descriptions (6898/23 621%‐29.2%) than those categorized as “intolerance” (583/3693%‐15.8%) (*P* < .0001).

Table 1 describes the profession of the health care worker entering in the adverse reaction information. Nursing staff entered the majority of reactions (60.1%), whereas pharmacists entered only 6.1%.

**Table 1:** Profession of clinician entering in adverse drug reaction information (Includes n = 12 755 cases where patients had greater than one allergen documented)

Professional	Number of entries	Percentage of total
Nurse	33 503	60.1%
Doctor	17 279	31.0%
Pharmacist	3384	6.1%
Allied health	921	1.7%
Student	608	1.1%
Clerical	71	0.1%

**Figure 3:**
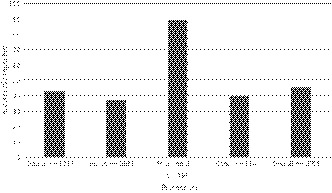
Percentage of adverse drug reactions described as one of; nausea, gastrointestinal upset, diarrhea, cramps, constipation, or headache, which were correctly categorized as intolerance by profession

There were 3705 ADR records using the six reaction terms consistent with intolerance, but only 1671 (45.1%) were correctly categorized as “intolerance.” Pharmacists were significantly more likely than other clinicians to correctly categorize these reactions as intolerance rather than allergy (*P* < .0001) (Figure 3).

Specific ADR scenario categorizations and comparison with survey results are listed in Table 2. All the intolerance reactions and Stevens‐Johnson syndrome were significantly more likely to be categorized as allergy in the EHR compared with the survey. There was a total of 4737 documented reactions to opioids, 3595 (75.9%) of which were categorized as allergy. Only 692 (14.6%) of these had a reaction type consistent with allergy, and of the remaining 4045, 2936 (72.5%) were intolerance reactions incorrectly categorized as allergy (Table 2). Almost half (1980, 49%) of these ADR were reported using the descriptor “other,” with a free text description of the reaction. ACE‐inhibitor associated angioedema was universally categorized as allergy although it is nonimmunological, although angioedema does not appear in the drop‐down list of reactions for intolerances. Anaphylaxis was correctly reported as allergy in 99.8% of cases, SJS was correctly categorized as allergy in all cases; both of these terms appear in the drop‐down list of reactions for allergy. Statin‐associated myalgia was most commonly categorized as allergy; as it did not appear in the allergy drop‐down list, it was entered as free text in these cases. Urticaria or rash secondary to NSAIDs, most commonly an intolerance, was categorized as allergy in almost all cases. Headache was categorized as allergy in more than half of cases.

**Table 2:** Categorization of clinical scenarios in the electronic health record (EPAS) compared with survey^1^

	n	Allergy	Intolerance	*P* value^a^
Anaphylaxis				
EHR	1281	*99.8%*	0.2%	.14
Survey	394	*98.5%*	0.8%	
Stevens‐Johnson syndrome				
EHR	29	*100.0%*	0.0%	.0008
Survey	394	*76.4%*	23.1%	
Headache				
EHR	212	55.0%	*45.0%*	<.0001
Survey	394	2.0%	*96.9%*	
Statin Myalgia				
EHR	101	63.0%	*37.0%*	<.0001
Survey	394	17.8%	*79.9%*	
ACE associated angioedema				
EHR	33	100.0%	*0.0%*	.0003
Survey	394	74.6%	*24.4%*	
NSAIDs urticaria/rash				
EHR	140	98.6%	*1.4%*	.0039
Survey	394	91.1%	*7.9%*	
True intolerance^b^				
EHR	3705	54.9%	*45.1%*	NA
Survey	*Not assessed*			
Opioid reactions excluding possible allergy^c^				
EHR	4045	78.5%	*27.5%*	NA
Survey	*Not assessed*			

Note

Items in italics denote correct categorization.

a Fisher's exact test denotes significant difference in categorization as allergy/intolerance dependent on whether data are from EHR or Survey.^1^

b Reactions described as; “nausea,” “gastrointestinal upset,” “diarrhea,” “cramps,” “constipation,” and “headache.”

c Reactions described as anaphylaxis, urticaria, shortness of breath, rash, serum sickness, swelling, and wheezing were excluded.

## 4 DISCUSSION

This study is an analysis of documentation of adverse drug reactions in an enterprise‐wide electronic health record across multiple sites, and by different clinician groups. We found that 15.6% of all patient records had at least one documented adverse reaction, the majority of which were adverse drug reactions (78.2%), with the most frequently reported drug types being antimicrobials, then opioids. The vast majority of drug names were chosen from the system's drug list (96.4%) permitting drug allergy crosschecks, in contrast to the large proportion of reaction descriptions utilizing free text to describe the adverse drug reaction (27.4% of total).

The principal finding of this study is that the vast majority (86.5%) of reactions documented were categorized as allergy. This level is not consistent with the general understanding that the majority of ADR are due to type A reactions.^5^ Even obvious intolerances such as “nausea,” “gastrointestinal upset,” “diarrhea,” “cramps,” “constipation,” and “headache” were incorrectly categorized as allergy in 54.9% of cases. This was independent of the profession of the HCW who entered the data, with the exception of pharmacists, who appear to document intolerances more accurately. The other principal finding was that for over a quarter of the reactions, “other” was selected from the reaction list, and the reaction description entered in as free text.

The commonest medications for which a reaction was documented were penicillins and opioids. The majority of these reactions were categorized as allergy (95% and 75%, respectively) even after reactions actually consistent with true allergy were removed from the analysis.^7^ Patients with an antimicrobial allergy label have worse hospital outcomes and more than 90% of patients with documented penicillin allergy are not found to be allergic on careful review.^8^, ^9^ There is evidence that even when allergy testing has been undertaken, allergy documentation is frequently not removed or is re‐entered into notes,^10^, ^11^ or clinicians override a drug allergy alert rather than update the documentation.^12^ Inaccurate documentation for opioids may result in excessive alerting when multiple opioids are prescribed, for example, in postoperative setting. This can result in alert fatigue, and the prescriber may miss other decision support alerts such as drug‐drug interaction or dose range alerts. Our study found that although details of opioid adverse reactions were often recorded, the majority (64.4%) did not select a pre‐specified ADR type but used the description of “other” then entered free text, which means that the information is more difficult to access for subsequent prescribers.

Our finding that intolerance is frequently miscategorized as allergy is not solely due to poor clinician understanding. Table 2 shows the rates of categorization in the EHR compared with the survey, there was a significant difference in miscategorization as allergy for all of the examples shown: headache, statin myalgia, ACE associated angioedema and NSAIDs associated urticaria. For true allergies such as SJS and anaphylaxis, the scenario‐based survey showed correct categorization in 75% and 99%, respectively, compared with 99.8% and 100% in electronic heath records, indicating the significant bias toward categorization as allergy in the EHR.

These findings taken together therefore provide evidence that the EHR interface influences the categorization of ADR. Terminology used in EHR is confusing, for example, all adverse reactions of any type are entered via an icon entitled “Allergies,” yet the user is then asked to classify the adverse reaction into allergy or intolerance. In the SA Health implementation of this EHR, allergy as a reaction type is defaulted. This is the likely explanation for our finding that the majority of reactions were selected as allergy, and that the rates of miscategorization of intolerances as allergy within the EHR was actually higher than in our clinician knowledge survey. This appeared to be the case for all clinicians except for pharmacists. Hence, it appears that although pharmacists have the same knowledge base regarding allergies and intolerances, compared to other clinicians, they are more careful when documenting these in an EHR. Unfortunately, in our population only 6% of all reactions were documented by pharmacists.

At the time of the audit, the configuration of the reaction list for allergy contained options generally consistent with immunological reactions eg anaphylaxis, SJS, angioedema, urticaria, and the list for intolerance contained nonimmunological side effects. However, these lists are available only after the reaction type, that is, allergy or intolerance has been selected. Furthermore, these lists are technically incorrect, such that, for example, angioedema is available only in the allergies list, but it is an adverse effect of the pharmacological action of ACE‐inhibitors, and is therefore, an intolerance. Urticaria is a nonimmunological effect of the histamine‐releasing activity of opiates and is also an intolerance. Therefore, the EHR does not allow correct mechanistic categorization of these reactions unless free text entries are used. Due to the defaulting of “allergy” as the category, and the absence of intolerance reactions in the subsequent reaction list, approximately a quarter of the reactions were documented as “other,” and the reaction was entered as free text in the description field. The influence of the default selection of “allergy” is such that clinicians were prepared to choose “other,” and add in additional text information, rather than having the extra initial click of choosing “intolerance” as the reaction type.

EHRs have the advantage of one‐time documentation of drug reaction information which is then available to all clinicians within the institution, and can be used for decision support to automatically generate customized prompts and alerts if the drug is prescribed again. Ideally, the prompt should be underpinned by a drug database, which takes into account medication cross‐reactivity, as clinicians are unable reliably know this information, and should ideally be tiered based on the risk of the adverse drug reaction upon re‐exposure; for example, nausea with opioids would warrant a different prompt to anaphylaxis with penicillins. The EHR also facilitates communication and dissemination of such information. In a previous study we found that EHR‐generated referral letters from general practitioners to specialists were far more likely to contain drug alerts than handwritten letters.^13^

The two main weak points in this system are the accuracy of information entered, and mechanistic interpretation by the HCW who is making the entry. The information entered is usually based on patient recall or existing documentation; there are reports of disparity between EHR documentation of adverse reactions and patient interviews*,* which also occurs with paper records.^14^, ^15^ We have shown here that mechanistic categorization is also faulty, and in our opinion given the large number and diversity of HCW making the entries, it is unlikely that further training in understanding the mechanisms of ADR to distinguish immunological from nonimmunological reactions would be helpful.

Our previous scenario‐based survey demonstrated that clinician understanding of ADR mechanistic categorization is poor. This study shows that this categorization is used poorly in practice. Furthermore, the EHR interface design may have an adverse influence on category selection. Taken together these findings lead us to suggest that ADR categorization as allergy or intolerance at the level of the ordinary HCW registering the patient in the EHR is of questionable usefulness. Indeed, a previous report from Spain on allergy documentation in an (unspecified) EHR pooled together allergies and intolerances since “both concepts were used indistinctly.”^16^ The obligatory classification of ADRs into allergy or intolerance at the time of entry is redundant since it provides no practical benefit, and may be counterproductive. Strengths of this study are the large number of reactions documented across an enterprise‐wide system. However, the potential weaknesses are that the reaction types were evaluated only for internal consistency but could not be validated against actual reactions, and that we have not had the opportunity to compare these conclusions against an alternative EHR which does not use obligatory classification.

In conclusion, categorization as allergy or intolerance is likely to be influenced by the EHR design, with the majority of ADRs categorized as allergy, even obvious intolerances. We suggest that it is more important that the description of the adverse reaction be entered as accurately as possible into the EHR, preferably at the time of occurrence or shortly afterwards. These details could then be available to inform future prescribers who could intelligently interpret the alert or seek assistance. This may then facilitate the development of intelligent decision support systems which are able to interpret likely mechanisms based on the nature of the reaction and the putative trigger and provide information on re‐exposure risk and cross‐reaction risk. The EHR interface design is a critical determinant of optimal risk‐stratified prescription alerting.

## AUTHOR CONTRIBUTIONS

Drs. Foreman, Smith, and Shakib conceptualized and designed the study. Drs Foreman and Caughey analyzed the data. All authors interpreted the data, reviewed and revised the manuscript. Drs Foreman and Caughey drafted the initial manuscript. All authors approved the final manuscript as submitted and agree to be accountable for all aspects of the work.

## DISCLOSURE AND ETHICS STATEMENT

No conflict of interest is reported by the authors. Ethics approval for this study was obtained from the South Australian Health Human Research Ethics Committee.

## Data Availability

Research data are not available for sharing.
